# Supplementary material for: Atco, a yeast mitochondrial complex of Atp9 and Cox6, is an assembly intermediate of the ATP synthase
Source: PLoS One. 2020 May 15;15(5):e0233177. doi: 10.1371/journal.pone.0233177 (PMC7228087; doi:10.1371/journal.pone.0233177)
Supplement: S1 Raw Images — (PDF) [file pone.0233177.s001.pdf]

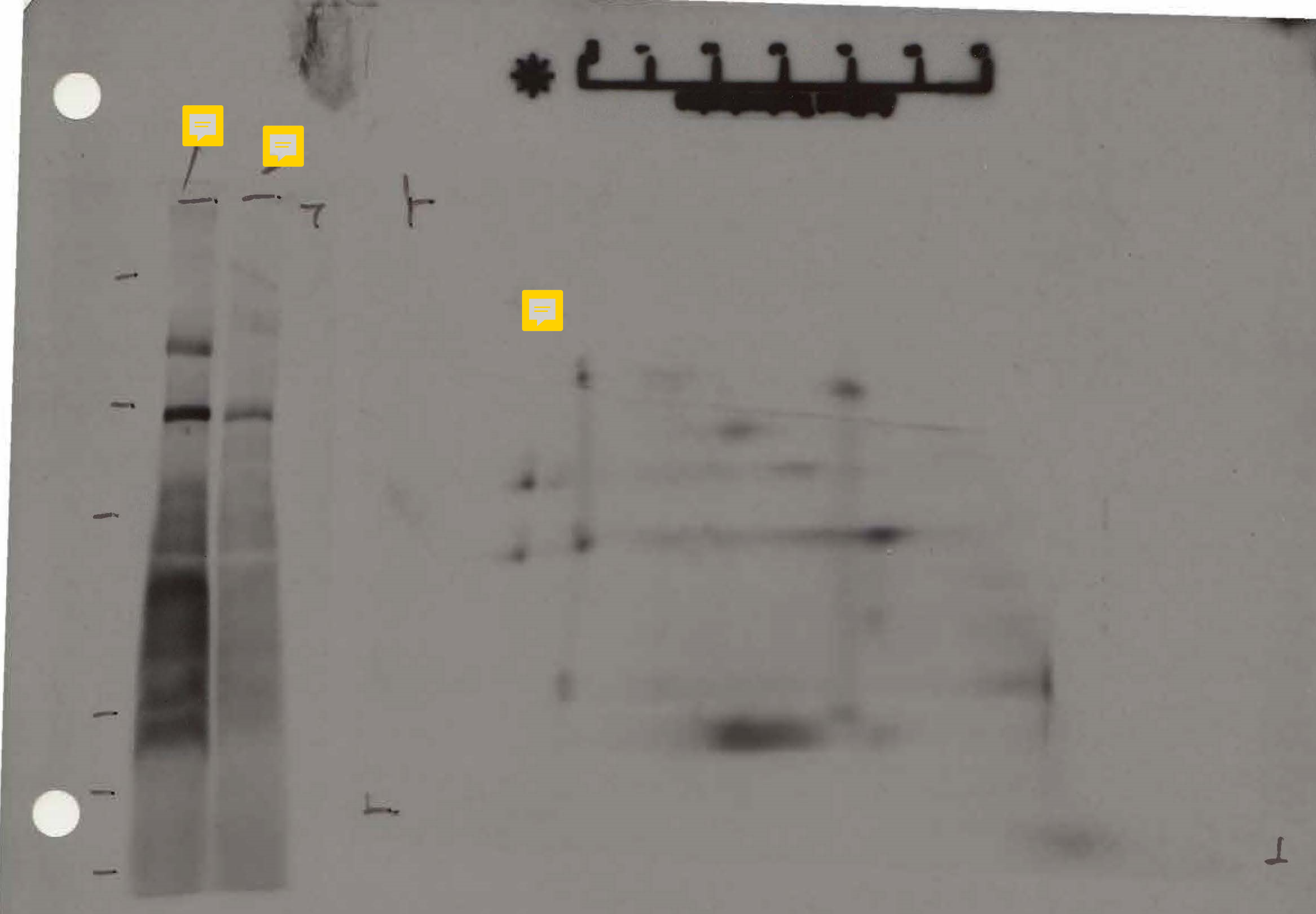

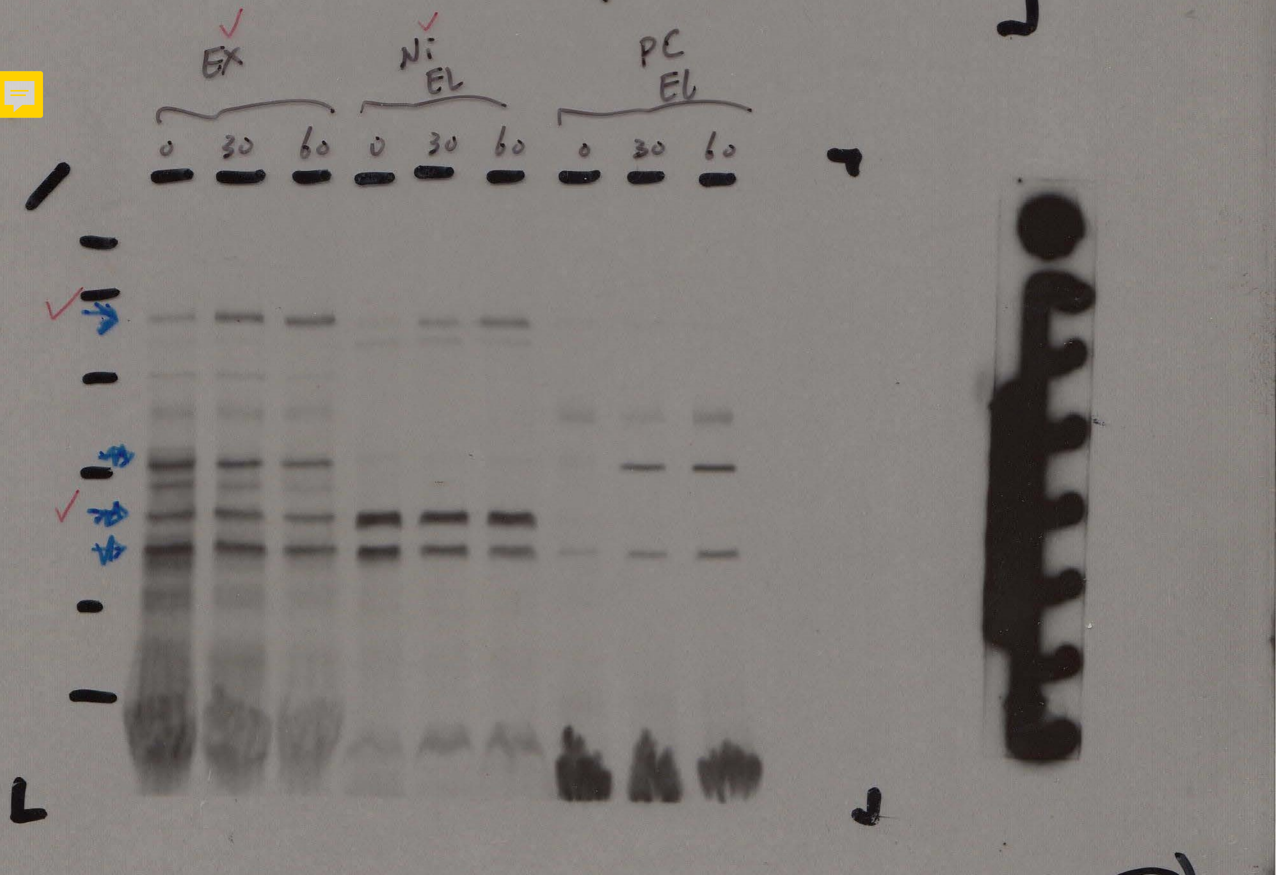

EX
Ni EL
PC El
0
30
60
0
30
60
0
30
60

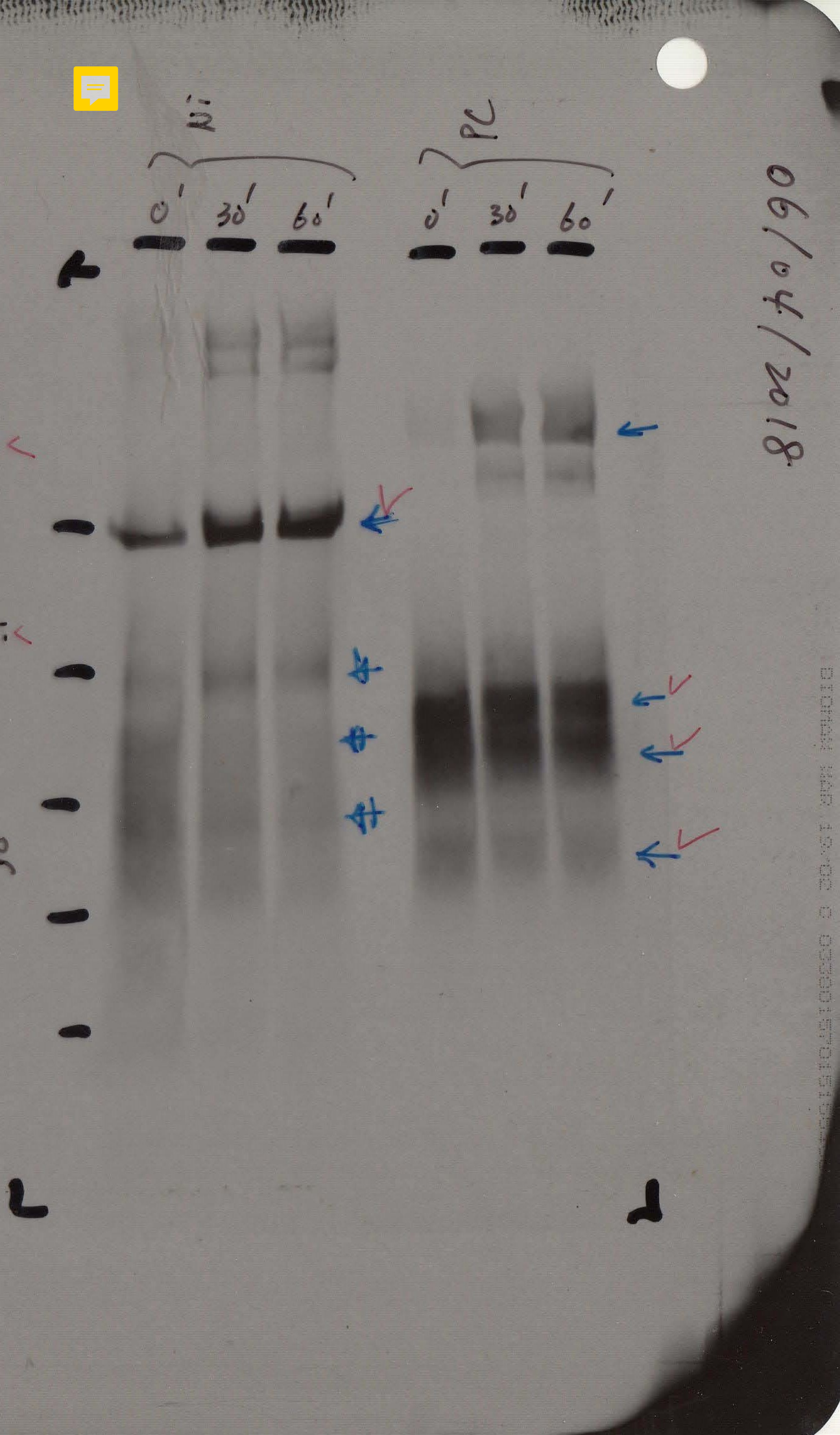
Ni
0'
30'
60'
PC
0'
30'
60'
06/04/2018

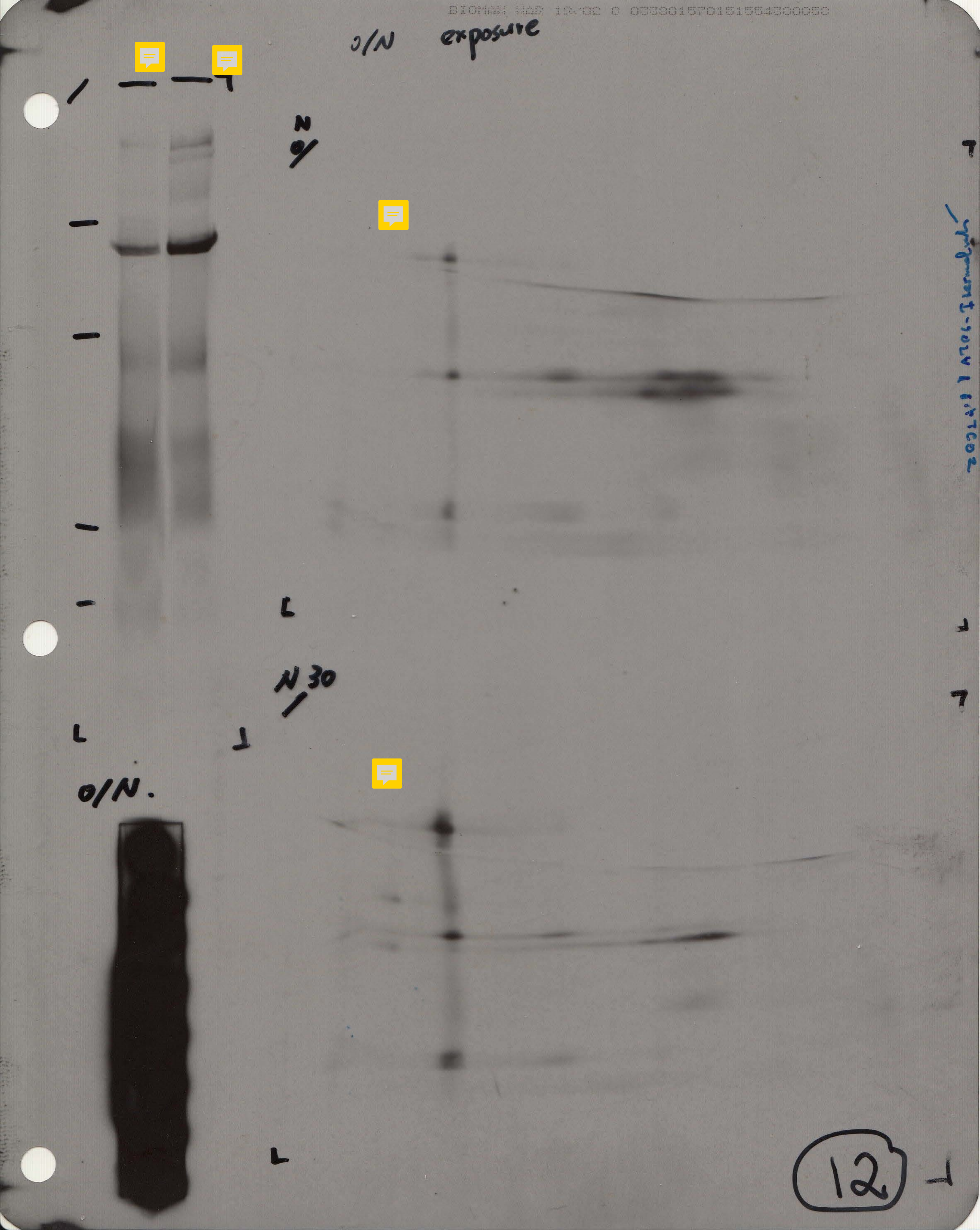
O/N exposure
N 0
N 30
O/N.
12

3/05/2019

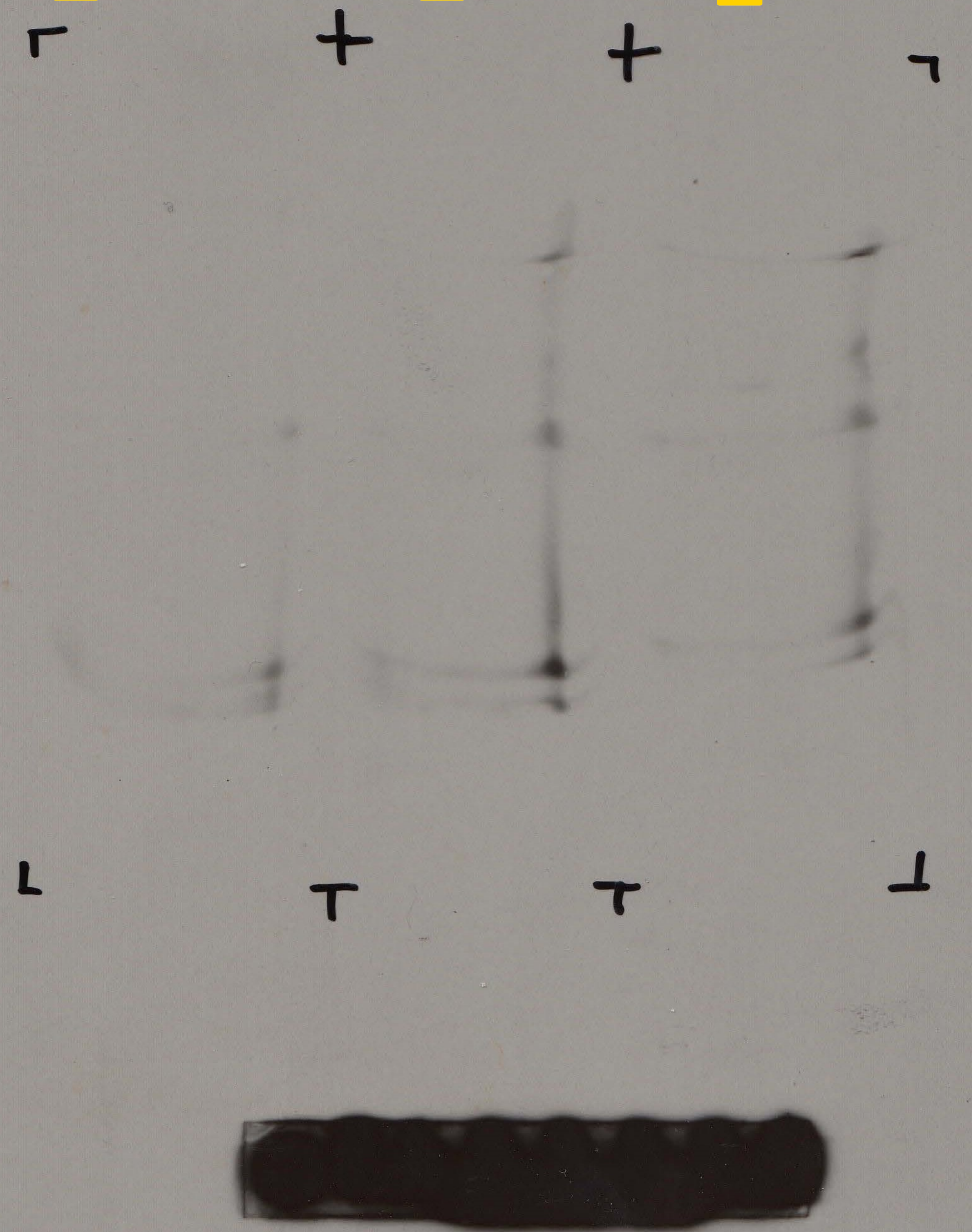

3 days.

(13)

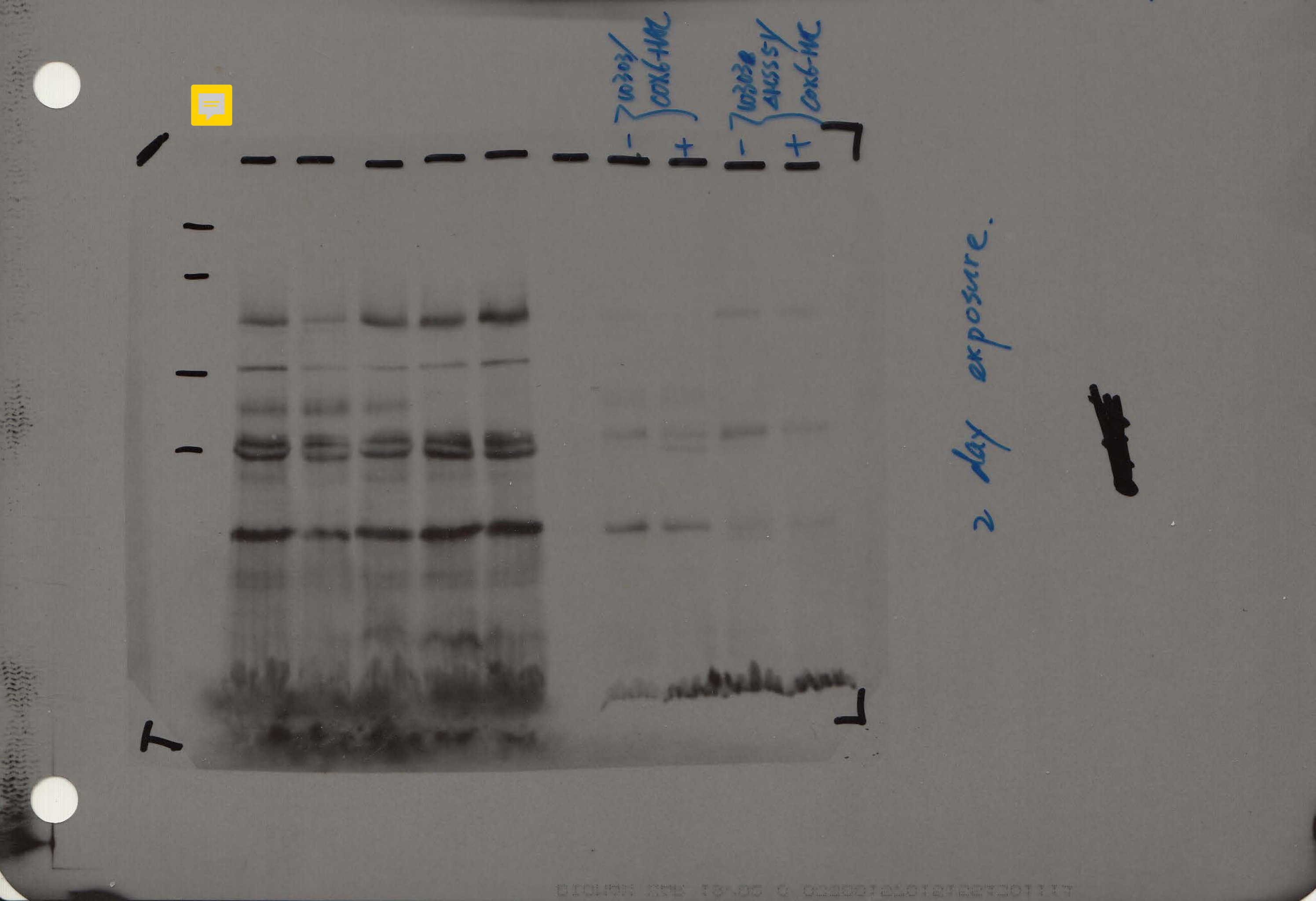
10303/
COX6-HAC
-
+
COX6-HAC
-
+
2 day exposure.

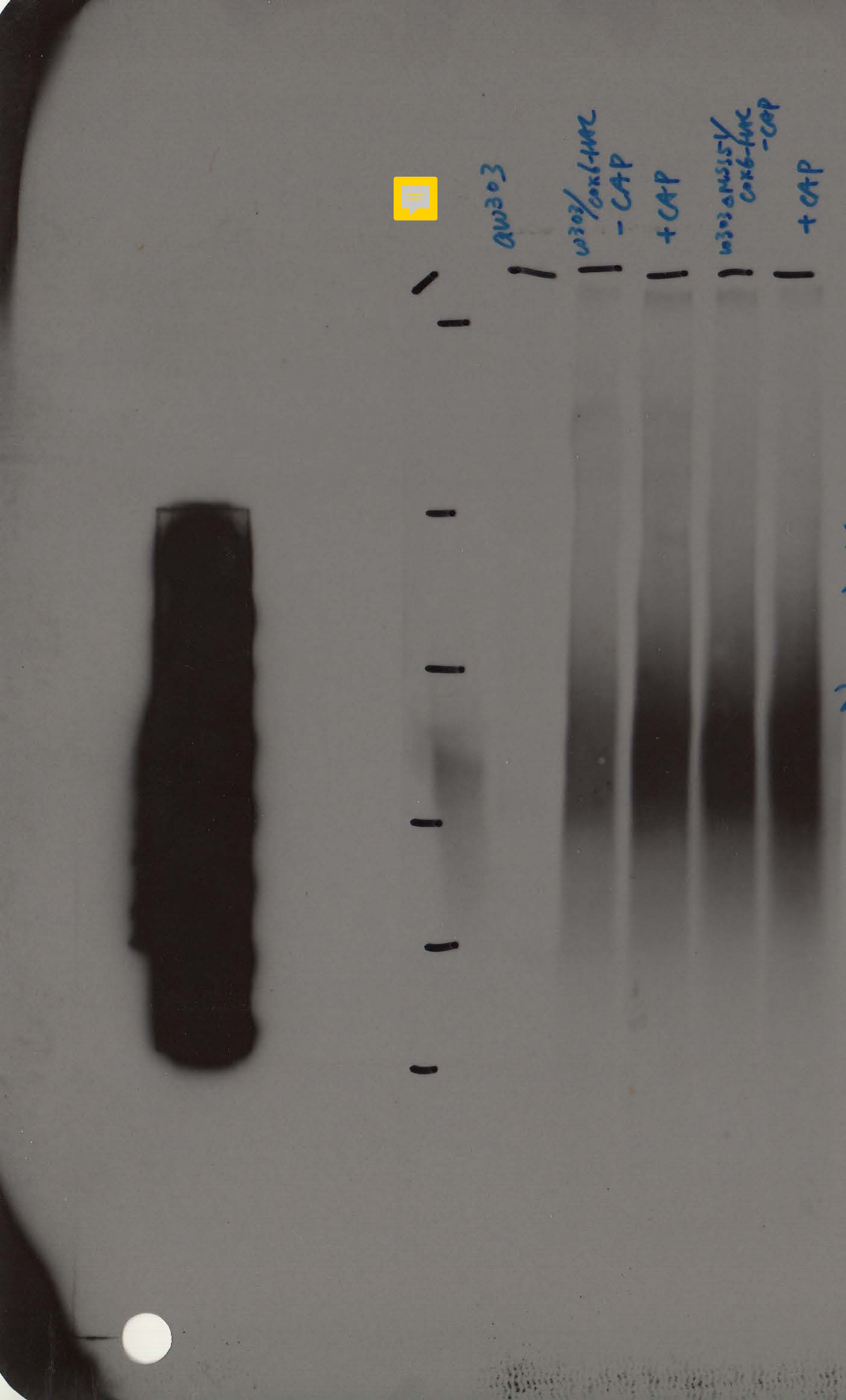
aW303
W303/cox6-HAC
- CAP
+ CAP
W303 ΔMSS51/cox6-HAC
- CAP
+ CAP

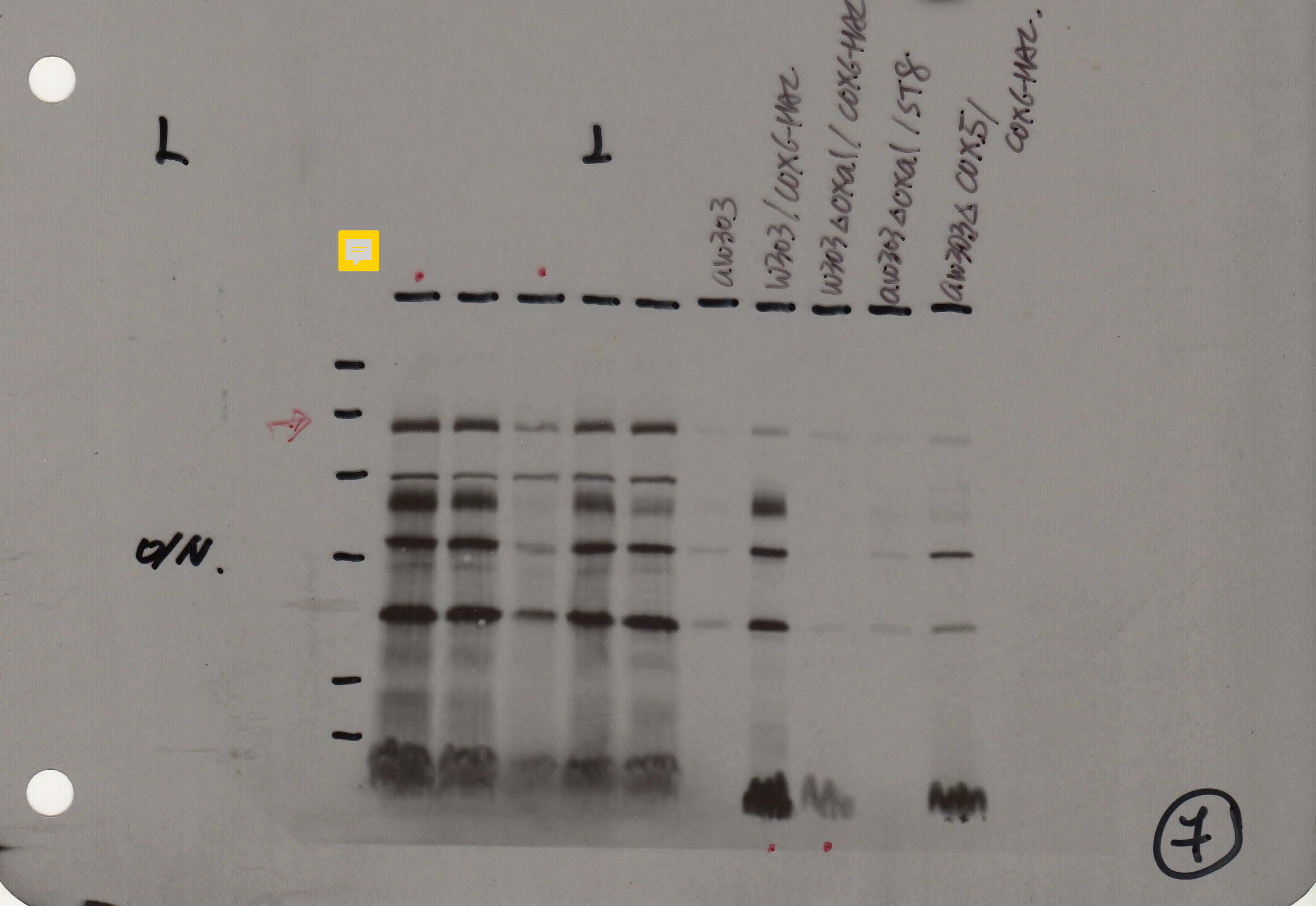

L
L
aW303
W303/COX6-HAC
W303ΔOXA1/COX6-HAC
aW303ΔOXA1/ST8
aW303ΔCOX5/COX6-HAC.
d/N.
7

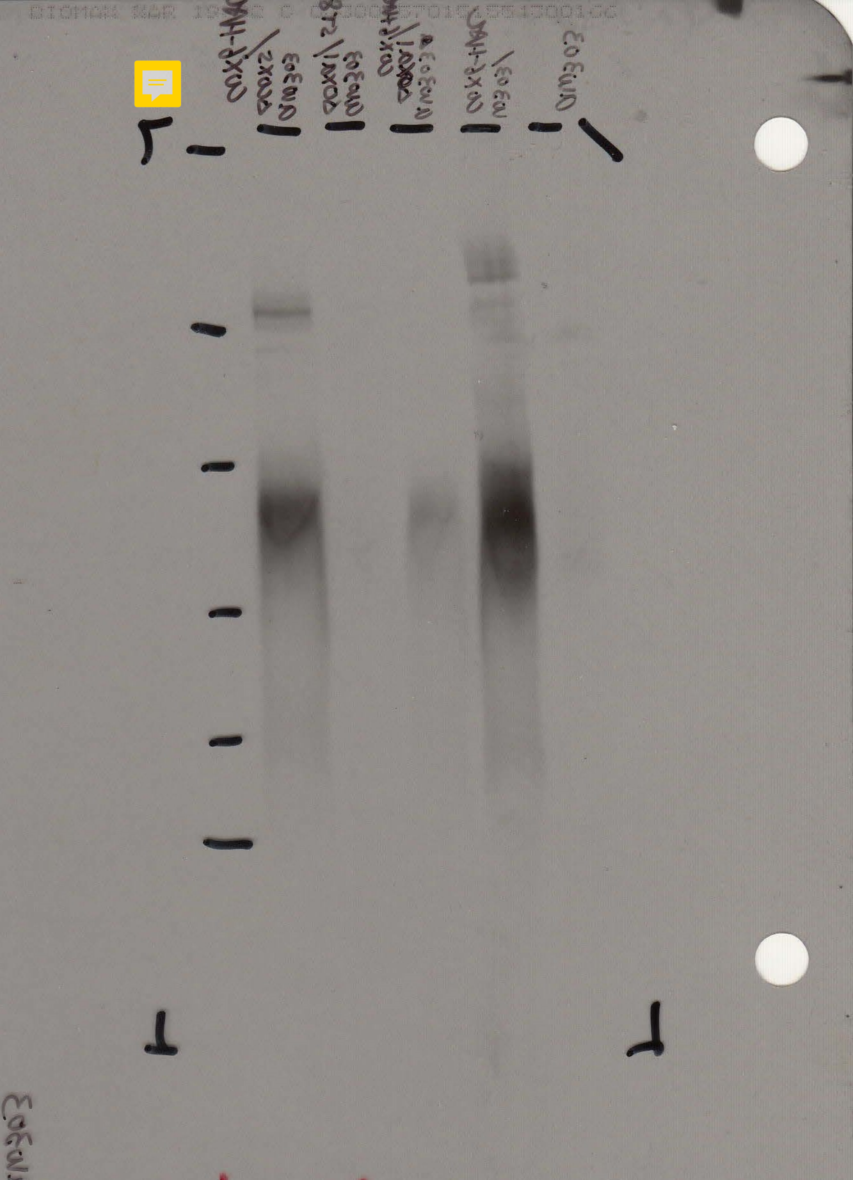

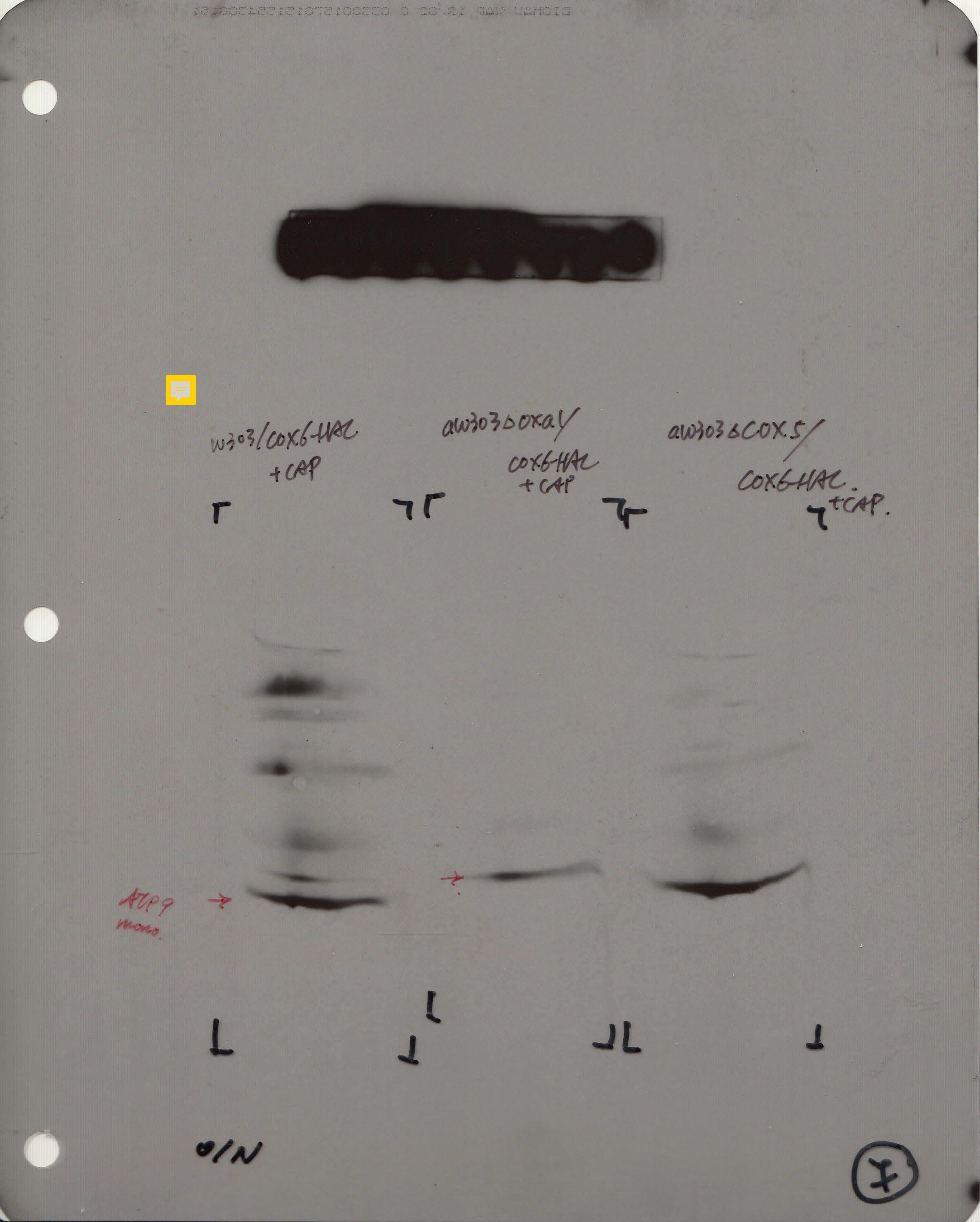
w303/COX6-HAC
+CAP
aW303ΔCOXa.1/
COX6HAC
+CAP
aW303ΔCOX5/
COX6HAC.
+CAP.
ATP9
mono.
O/N

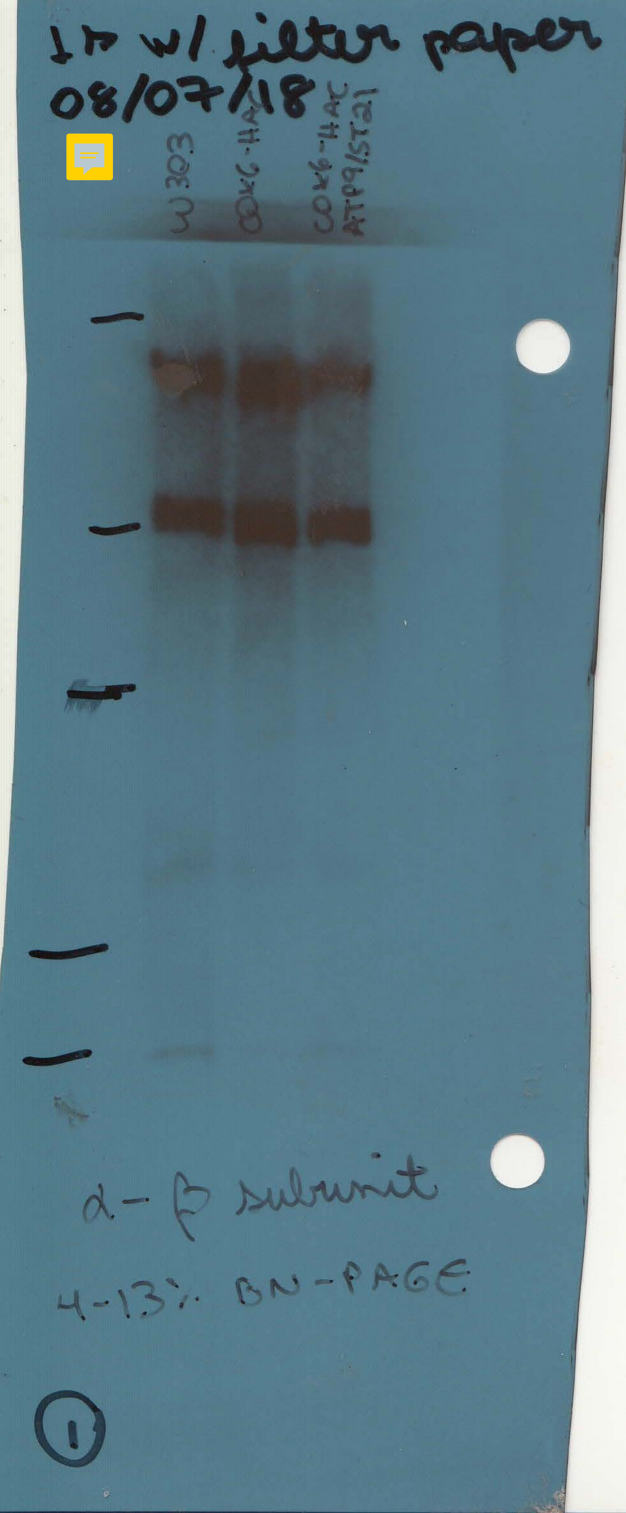
1h w/ filter paper
08/07/18
W303
COX6-HAC
COX6-HAC ATP9/ST21
α- β subunit
4-13% BN-PAGE
1

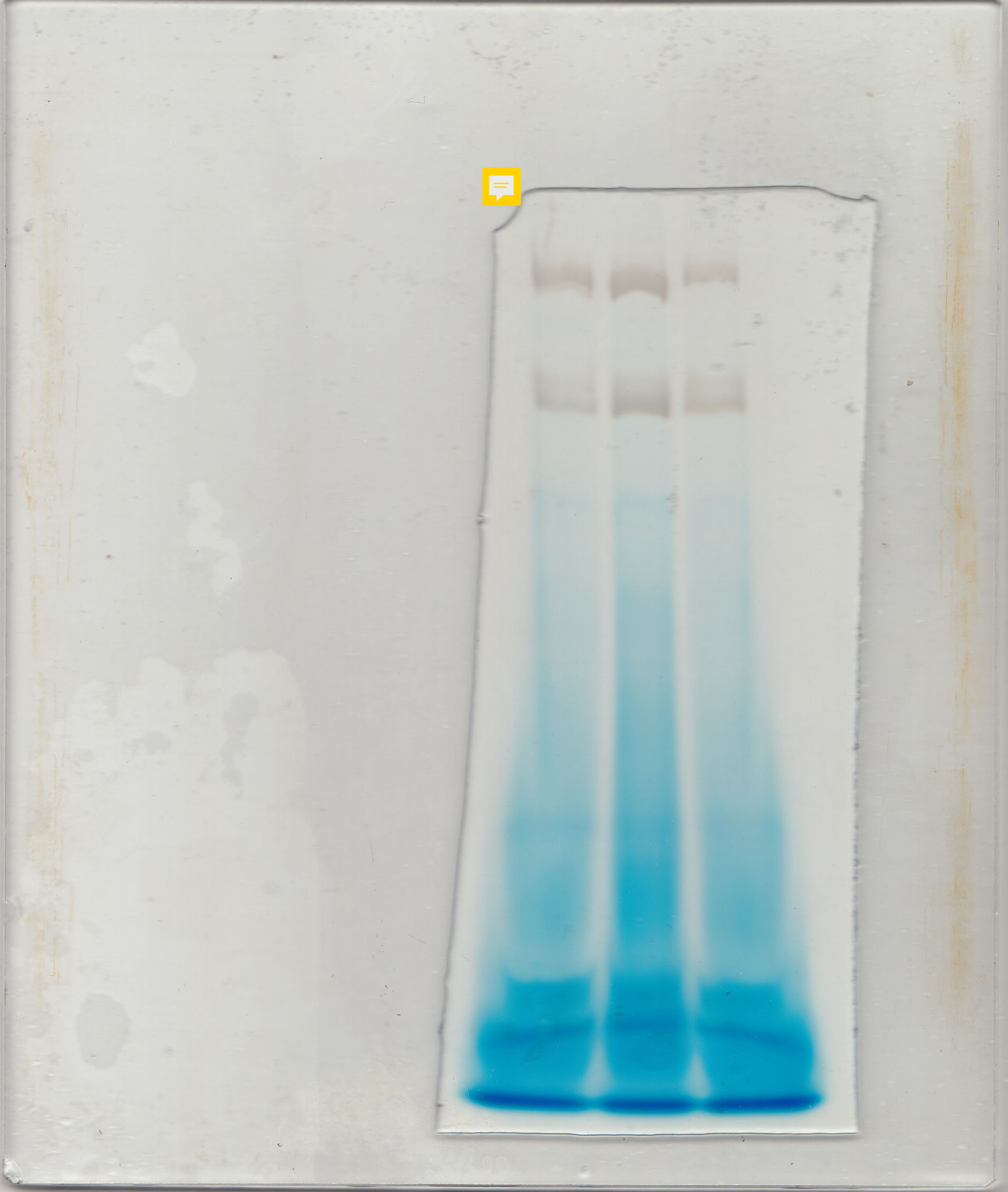

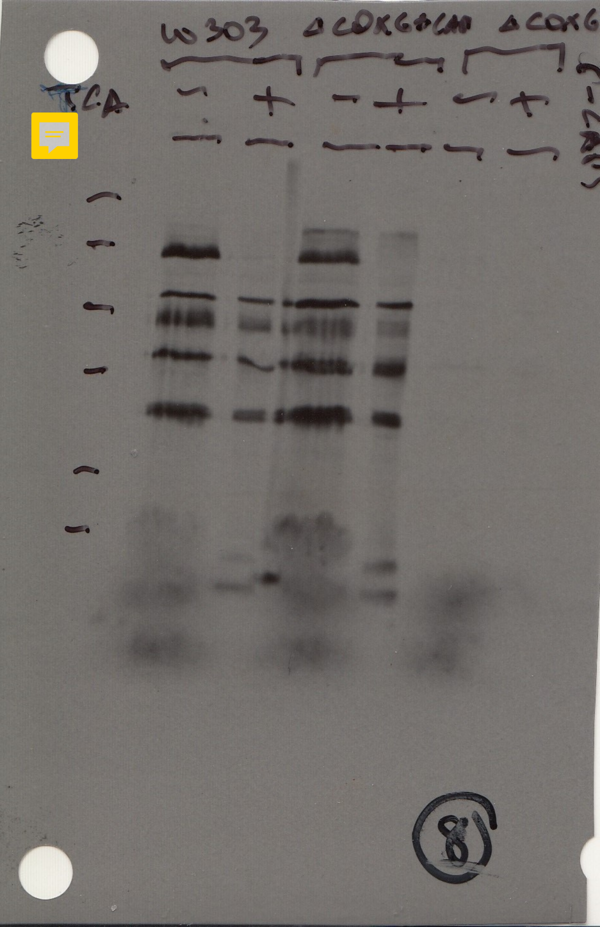
w303
ΔCOX6+CAP
ΔCOX6
TCA
- + - + - +
81

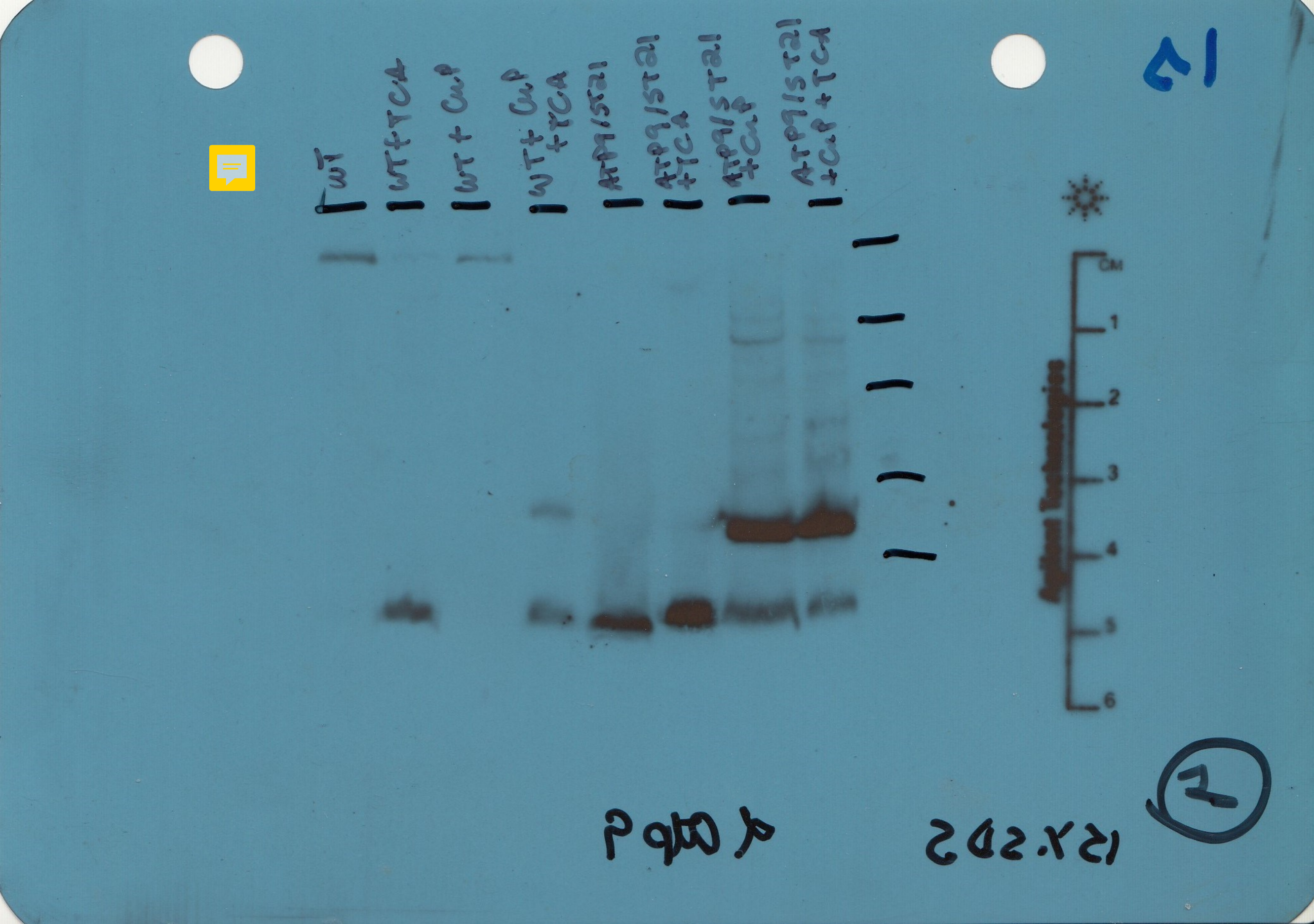
15
WT
WT+TCA
WT + CuP
WT + CuP + TCA
ATP9/5T21
ATP9/5T21 +TCA
ATP9/5T21 +CuP
ATP9/5T21 +CuP+TCA
CM
1
2
3
4
5
6

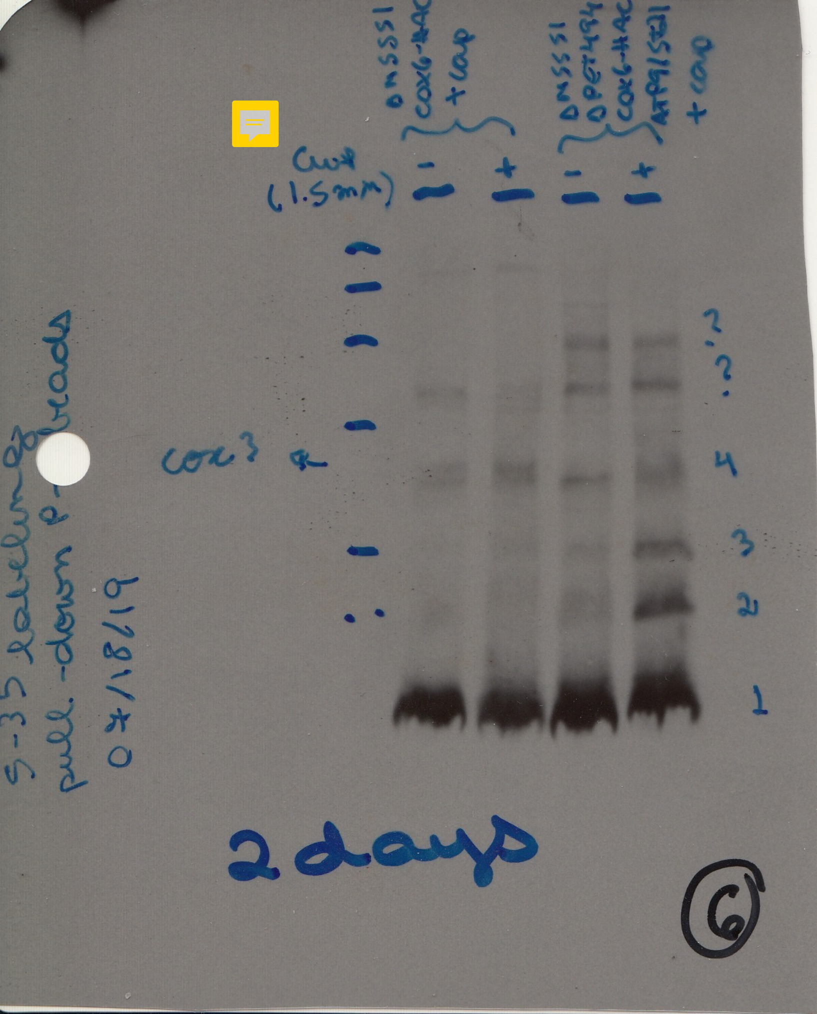

S-35 labeling
pull.-down P-beads
07/18/19
Cox3
Cu (1.5mM)
- +
ΔMSS51 COX6-HAC + COP
ΔMSS51 ΔPET494 COX6-HAC ATP9/ST21 + COP
1
2
3
4
2 days
6

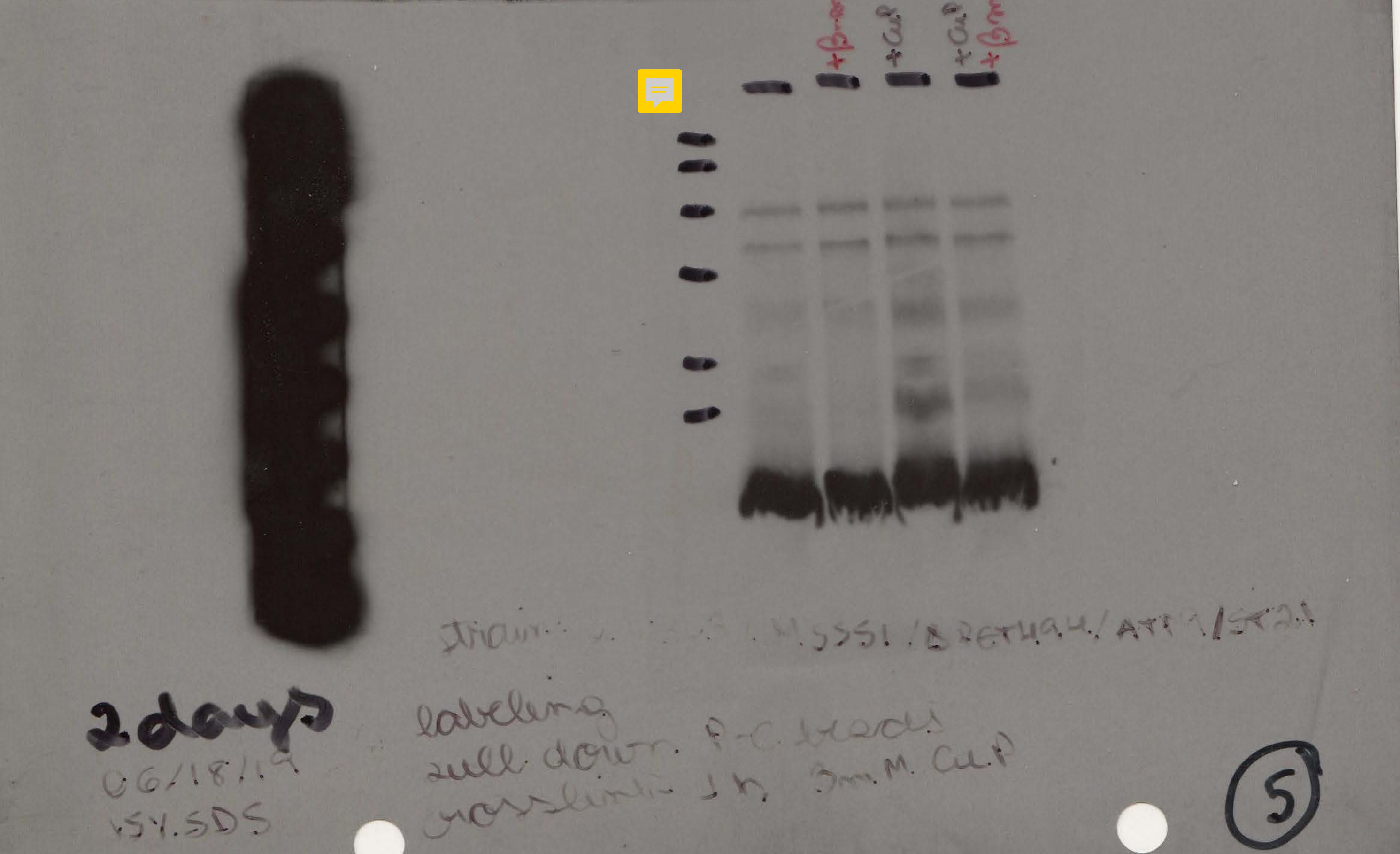
+CuP
2days
06/18/19
15%.SDS
labeling
5
